# Magnetoencephalographic Study on Forward Suppression by Ipsilateral, Contralateral, and Binaural Maskers

**DOI:** 10.1371/journal.pone.0066225

**Published:** 2013-06-06

**Authors:** Tadashi Nishimura, Yuka Uratani, Tadao Okayasu, Seiji Nakagawa, Hiroshi Hosoi

**Affiliations:** 1 Department of Otolaryngology-Head and Neck surgery, Nara Medical University, Nara, Japan; 2 Health Research Institute, National Institute of Advanced Industrial Science and Technology (AIST), Ikeda, Osaka, Japan; Oregon Health & Science University, United States of America

## Abstract

When two tones are presented in a short time interval, the response to the second tone is suppressed. This phenomenon is referred to as forward suppression. To address the effect of the masker laterality on forward suppression, magnetoencephalographic responses were investigated for eight subjects with normal hearing when the preceding maskers were presented ipsilaterally, contralaterally, and binaurally. We employed three masker intensity conditions: the ipsilateral-strong, left-right-balanced, and contralateral-strong conditions. Regarding the responses to the maskers without signal, the N1m amplitude evoked by the left and binaural maskers was significantly larger than that evoked by the right masker for the left-strong and left-right-balanced conditions. No significant difference was observed for the right-strong condition. Regarding the subsequent N1m amplitudes, they were attenuated by the presence of the left, binaural, and right maskers for all conditions. For the left- and right-strong conditions, the subsequent N1m amplitude in the presence of the left masker was smaller than those of the binaural and right maskers. No difference was observed between the binaural and right masker presentation. For left-right-balanced condition, the subsequent N1m amplitude decreased in the presence of the right, binaural, and left maskers in that order. If the preceding activity reflected the ability to suppress the subsequent activity, the forward suppression by the left masker would be superior to that by the right masker for the left-strong and left-right-balanced conditions. Furthermore, the forward suppression by the binaural masker would be expected to be superior to that by the left masker owing to additional afferent activity from the right ear. Thus, the current results suggest that the forward suppression by ipsilateral maskers is superior to that by contralateral maskers although both maskers evoked the N1m amplitudes to the same degree. Additional masker at the contralateral ear can attenuate the forward suppression by the ipsilateral masker.

## Introduction

Brain responses to multiple tones differ from those to single tone. In the presentation of multiple tones, time lag among the stimuli also influences the neural activity. When two tones are presented in a short time interval, the response to the second tone is suppressed [1], [2], [3]. This phenomenon is referred to as forward suppression. While forward suppression has not been fully elucidated, various influential factors have been revealed by numerous studies that employed physiologic methods in animals [1], [2], [3]. On the other hand, not so many studies using electroencephalography, magnetoencephalography (MEG) and functional MRI in human have been performed.

A previous MEG study demonstrated that the interval between two tones influences the suppression of the subsequent N1m amplitude [4]. It is reasonable to consider that the forward suppression is elevated as the interval is shorter. However, for intervals <40 ms, the subsequent N1m amplitude increased as the interval became shorter. Such increase for short intervals has not been observed in previous animal studies [1], [2]. Considering the effect of signal duration on the N1 or N1m amplitudes, the temporal window at the N1m level is estimated within approximately 40 ms [5], [6], [7]. Presenting two tones at the intervals below 40 ms, both neural inputs from the peripheral are simultaneously involved in the temporal window, which may evoke additional activity at the N1m level. This might lead to the elevation of the N1m amplitude. The suppression of MEG responses in human are also affected by other factors which have not been observed for physiologic studies in animals.

In the peripheral nervous system, forward suppression is induced by an ipsilateral masker, and neural adaptation is considered as the mechanism underlying the suppression [8]. In the central nervous system, inhibitory neural interaction also participates in the suppression [1], [2], [3]. Because ascending auditory pathways from both ears have interactions with each other beyond the brain stem, the suppression can be induced not only by ipsilateral masker but also by the contralateral masker. However, the difference in forward suppression among the presence of ipsilateral, contralateral, and binaural maskers has not been revealed. In this study, the forward suppressions by these three maskers were evaluated using MEG. As mentioned above, the interval between two tones has a great influence on suppression, and involvement of both neural inputs in the same temporal window would complicate interpretation. Consequently, the interval of 40 was chosen, as the maximum significant suppression was observed in the previous study [4]. Furthermore, the influence of the masker-intensity balance between both ears on forward suppression was also investigated in this study.

## Materials and Methods

Eight volunteers with normal hearing (4 females and 4 males, 24–30 years old) participated in this study. All the subjects were right handed. This study was approved by the ethical committee of the National Institute of Advanced Industrial Science and Technology. Participants provided written informed consent before being enrolled.

**Figure 1:**
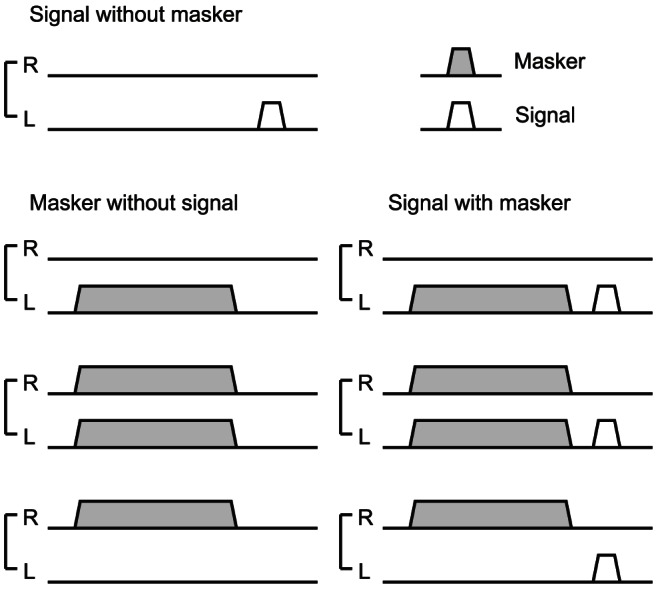
Schema of the seven stimulus sets. The presentations in the right (R) and left (L) ears are separately illustrated. The solid and open trapezoids indicate the masker and signal presence, respectively.

Two tones were presented in order. Tone bursts of 1 kHz were employed as both the preceding (masker) and subsequent (signal) tones. The duration of the masker and signal were 300 and 50 ms, including rise and fall ramps of 10 ms, respectively. The interval between the masker offset and the signal onset was 40 ms. The signal was presented to the left ear. The maskers were presented to the left ear (ipsilateral suppression), the right ear (contralateral suppression), and both ears (binaural suppression). Seven stimulus sets were given as follows: (1) signal without masker, (2–4) left, right, and binaural maskers without signal, and (5–7) signals with the left, right, and binaural maskers (Figure 1). The stimulus sets were randomly presented at intervals of 2.0±0.1 s. The measurements were done in three separate settings A, B and C. The left masker was set at 80 dB SPL and the right masker at 60 dB SPL in A, and vice versa in C. Both the left and right maskers were set at 60 dB SPL in B. The settings A, B and C were named as left-strong, left-right-balanced, and right-strong condition, respectively. The signal intensity was 60 dB SPL in common for the three conditions. The sounds were emitted by earphones (E-A-R TONE 3A; Cabot Safety Co., Indianapolis, IN) and delivered to the ears through a plastic tube. The earphones were calibrated with an ear simulator (Type 4157; Brüel & Kjær, Nærum, Denmark).

Magnetic responses evoked by the masker and the signal were recorded using a neuromagnetometer (Neuromag-122; Neuromag Ltd., Helsinki, Finland) in a magnetically shielded room. During the experiment, the subjects watched a self-chosen movie without any sound and were instructed to pay no attention to the stimuli. The magnetic data were sampled at 0.4 kHz after being band pass-filtered between 0.03 and 100 Hz and were then averaged more than 100 times. Any responses coinciding with the magnetic signals exceeding 3000 fT/cm were rejected from further analysis. The averaged responses were digitally band pass-filtered between 0.1 and 30 Hz. The analysis time was 1.0 s from 0.2 s prior to the stimulus onset. The average 0.2-s prestimulus period served as the baseline.

We identified the N1ms in the right hemisphere, and compared the peak amplitudes at the channel where the largest N1m amplitude was evoked by the signal without masker. The neuromagnetometer had two pick-up coils at each position, which measured the two tangential derivatives, *δ* ***B*** *_z_*/*δx* and *δ* ***B*** *_z_*/*δy*, of the field component ***B*** *_z_*. Thus, we determined:
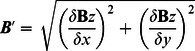



First, the responses evoked by the left, right, and binaural masker stimuli were compared. The N1m amplitudes evoked by the three maskers without signal were normalized to those evoked by the left masker without signal. The N1m amplitudes were compared every intensity condition. The data were analyzed using a one-way analysis of variance (ANOVA), with presentation laterality (left, right, and binaural) as within-subject factors. The Ryan method was used for post-hoc comparisons.

Second, in order to compare the forward suppression by the three maskers among the subjects, the N1m amplitudes evoked by the signal were normalized to the amplitude evoked by the signal without masker. Furthermore, the N1m amplitudes were also analyzed after removal of the responses to the maskers in order to eliminate the influence of the overlapping of the responses to the maskers. These values were calculated by the square means of the responses to the signal with the maskers after subtracting the response to the respective maskers without signal. The obtained values were also normalized to the N1m amplitude evoked by the signal without masker. The N1m amplitudes were compared every intensity condition. The data were analyzed using a one-way ANOVA, with masker condition (without masker, with left masker, right masker, and binaural masker) as within-subject factors. The Ryan method was used for post-hoc comparisons.

## Results

**Figure 2:**
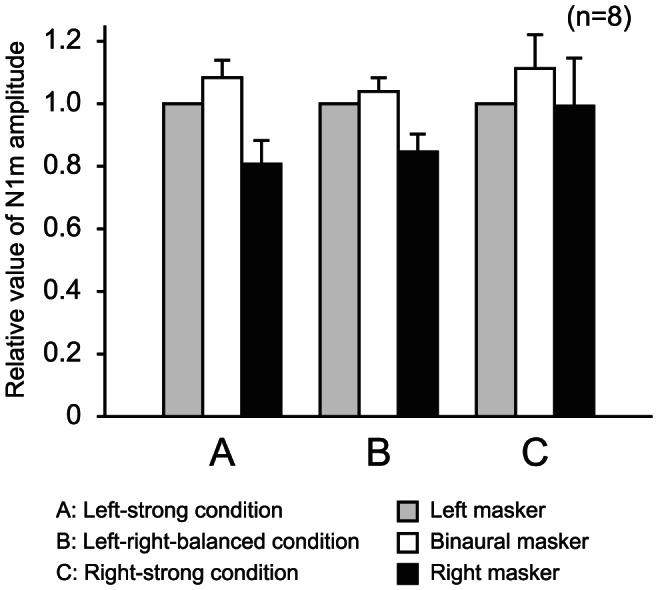
Mean N1m amplitudes evoked by three maskers without signal in three intensity conditions. The error bars indicate standard errors. Because the N1m amplitudes were normalized to those evoked by the left masker without signal in each intensity condition, the standard errors for the left masker without signal resulted in zero.

Figure 2 shows the mean N1m amplitudes evoked by the three maskers without signal. One-way ANOVAs revealed a statistically significant effect for presentation laterality for the left-strong (*F* [2], [14] = 5.525, *p*<0.05) and left-right-balanced conditions (*F* [2], [14] = 8.457, *p*<0.01). In multiple comparisons, the N1m amplitude evoked by the left and binaural maskers was significantly larger than that evoked by the right masker for the left-strong and left-right-balanced conditions (*p*<0.05). For the right-strong condition, no significant difference was observed.

**Figure 3:**
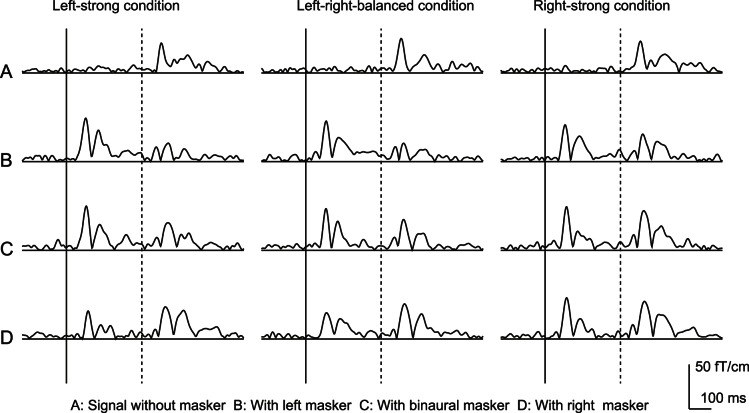
Square means of the responses at the channel where the maximum N1m amplitude was observed in a subject. The N1m amplitudes evoked by the signal decreased in the presence of the maskers. Solid and dashed vertical bars indicate the onset of masker and signal, respectively.

**Figure 4:**
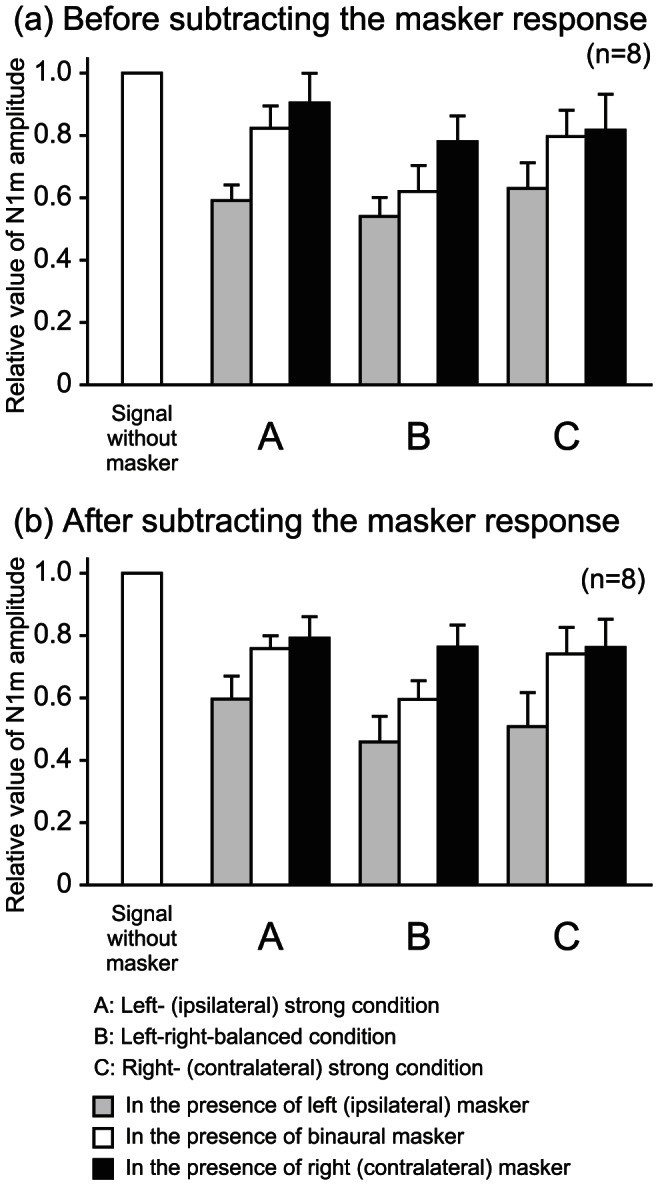
Mean N1m amplitudes normalized to the amplitude evoked by the signal without masker in three masker intensity conditions. The upper (a) and lower (b) graphs show the respective values before and after subtracting the masker response. The error bars indicate standard errors.

Figure 3 shows the square means of the responses at the channel where the maximum N1m amplitude was observed for a subject. The subsequent N1m amplitudes that were the responses to the signal were suppressed in the presence of the maskers for any conditions. Figure 4(a) shows the mean subsequent N1m amplitude before subtracting the responses to the maskers. One-way ANOVAs revealed a statistically significant effect for masker condition for all three conditions: the left-strong (*F*[3,21] = 65.811, *p*<0.01), left-right-balanced (*F*[3,21] = 52.8, *p*<0.01), and right-strong conditions (*F*[3,21] = 33.784, *p*<0.01). The subsequent N1m amplitudes were significantly attenuated by the presence of the left, binaural, and right maskers for all conditions (*p*<0.05). For the left- and right-strong conditions, the subsequent N1m amplitude in the presence of the left masker was significantly smaller than those of the binaural and right maskers (*p*<0.05). No difference was observed between the binaural and right masker presentation. For left-right-balanced condition, the subsequent N1m amplitude significantly decreased in the presence of the right, binaural, and left maskers in that order (*p*<0.05).

Figure 4(b) shows the mean subsequent N1m amplitude after subtracting the responses to the maskers. One-way ANOVAs also revealed a statistically significant effect for masker condition for all three conditions: the left-strong (*F*[3,21] = 36.229, *p*<0.01), left-right-balanced (*F*[3,21] = 40.163, *p*<0.01), and right-strong conditions (*F*[3,21] = 29.331, *p*<0.01). The subsequent N1m amplitudes were significantly attenuated by the presence of the left, binaural, and right maskers. For the left-strong condition, the subsequent N1m amplitude in the presence of the left masker was significantly smaller than that of the binaural and right maskers (*p*<0.05). No difference was observed between the binaural and right masker presentation. For the left-right-balanced condition, the subsequent N1m amplitude in the presence of the left and binaural maskers was significantly smaller than that of the right masker (*p*<0.05). For the right-strong condition, no significant difference was observed among the subsequent N1m amplitudes in the presence of the left, binaural, and right maskers.

## Discussion

Previous studies demonstrated the contralateral dominance for cortical auditory processing [9], [10], [11], [12]. Left and right stimuli can induce larger activities in the right and left hemispheres, respectively. This contralateral dominance probably leaded to the current results that the N1m amplitudes evoked by the left masker were larger than those evoked by the right masker in the right hemisphere for the left-strong and left-right-balanced conditions. For the right-strong condition, no difference was observed between the N1m amplitudes evoked by the right and left maskers. Generally, the N1m amplitudes depend on various factors such as intensity, duration, and frequency [7], [13], [14], [15]. Because an increase in intensity can elevate the N1m amplitude [13], [14], [15], the right-strong condition probably canceled the disadvantage due to the contralateral dominance.

The activities in the right and left cochlea are transmitted via the respective ascending auditory pathway. The power of the afferent activity spike arriving in the hemisphere for binaural presentation might be larger than that for monaural presentation. For the left-strong and left-right-balanced conditions, the mean N1m amplitude evoked by the binaural masker without signal was slightly larger than that evoked by the left masker without signal (Figure 2). For the right-strong condition, the mean N1m amplitude evoked by the binaural masker without signal was slightly larger than that evoked by the right masker without signal (Figure 2). However, no significant differences were recognized in these comparisons. The afferent activities from both ears are integrated [16], [17]. Neural interactions between them sometimes suppress the other auditory pathway [18]. Therefore, significant growth of the N1m amplitudes by binaural stimuli was not observed in the results.

If the preceding activity reflected the ability to suppress the subsequent activity, the forward suppression by the left masker would be superior to that by the right masker for the left-strong and left-right-balanced conditions, and no difference would be observed between them for the right-strong condition. In the current results, the forward suppression by the left masker was however larger than that by the right masker for all conditions. The preceding activity did not always reflect the forward suppression at the N1m level. The current results suggested that the forward suppression by ipsilateral maskers is superior to that by contralateral maskers although both maskers evoke the N1m amplitudes to the same degree.

The forward suppression by the binaural masker was expected to be superior to that by the left masker owing to additional afferent activity from the right ear. However, the results revealed the superiority of the left masker in forward suppression over the binaural masker. Particularly, for the right-strong condition, the forward suppression by the binaural masker was almost equal to that by the right masker. These findings suggested that the forward suppression by the ipsilateral masker is attenuated by the contralateral masker presence. The neural interactions between both left and right auditory pathways may attenuate the forward suppression.

## Conclusion

The forward suppressions by ipsilateral, contralateral, and binaural maskers were compared at the N1m level. The forward suppression by ipsilateral maskers is superior to that by contralateral maskers although both maskers evoke the N1m amplitudes to the same degree. Additional masker at the contralateral ear can attenuate the forward suppression by the ipsilateral masker.
